# Sympathetic Neurotransmitters and Tumor Angiogenesis—Link between Stress and Cancer Progression

**DOI:** 10.1155/2010/539706

**Published:** 2010-05-20

**Authors:** Jason Tilan, Joanna Kitlinska

**Affiliations:** Department of Physiology & Biophysics, Georgetown University, Basic Science Building 231A, 3900 Reservoir Rd., NW, Washington, DC 20007, USA

## Abstract

Recent evidence supports a longstanding hypothesis that chronic stress can influence tumor growth and progression. It has been shown that sympathetic neurotransmitters, such as catecholamines and neuropeptides, can affect both cancer cell growth and tumor vascularization. Depending on neurotransmitter and type of tumor, these effects can be both stimulatory and inhibitory. Norepinephrine (NE) and epinephrine (E) are potent stimulators of vascularization, acting both by inducing the release of angiogenic factors from tumor cells and directly on endothelial cell (EC) functions. As a result, activation of the adrenergic system increases growth of various types of tumors and has been shown to mediate stress-induced augmentation of tumor progression. Dopamine (DA), on the other hand, interferes with VEGF signaling in endothelial cells, blocks its angiogenic functions and inhibits tumor growth. Another sympathetic neurotransmitter coreleased with NE, neuropeptide Y (NPY), directly stimulates angiogenesis. However, proangiogenic actions of NPY can be altered by its direct effect on tumor cell proliferation and survival. In consequence, NPY can either stimulate or inhibit tumor growth, depending on tumor type. Hence, sympathetic neurotransmitters are powerful modulators of tumor growth and can become new targets in cancer therapy.

## 1. Introduction

Stress is an inevitable element of our lives. Stressful events activate the sympathetic nervous system and hypothalamic-pituitary-adrenal axis, which lead to the release of biochemical mediators of stress, such as cortisol, catecholamines, and neuropeptides [1, 2]. The elevated levels of these factors are used as clinical markers of stress. These stress mediators trigger a variety of physiological changes meant to improve the performance of the organism, such as increasing blood pressure and heart rate and enhancing the immune response. Thus, a short, acute stress has been shown to exert various beneficial effects. However, when stress becomes chronic, the prolonged exposure to the same stress mediators, which were beneficial in acute stress, often triggers pathological processes and contributes to the development or exacerbation of various diseases, including cancer [3].

 Chronic stress has been implicated in the stimulation of tumor development and progression by both clinical and animal studies [4–6]. Initially, stress-induced suppression of the immune response was suggested as the major mechanism of this phenomenon [7]. As opposed to acute stress, which enhances immunity and has been shown to increase resistance to cancer, chronic stress impairs immune responses and in this way facilitates tumor growth [8, 9]. However, there is also growing evidence indicating that stress mediators, such as glucocorticoids and sympathetic neurotransmitters, can directly affect tumor cell proliferation and survival as well as tumor angiogenesis. The direct effects on tumor cells vary significantly between different stress mediators and types of tumors [10–13]. In contrast, their actions on tumor vascularization involve interactions with common angiogenic factors, such as vascular endothelial growth factor (VEGF), and seem to be universal between different tumor types [5, 14–16]. Thus, stress mediators and their receptors can become novel targets in antiangiogenic tumor therapy. This review will focus on sympathetic neurotransmitters and their effects on tumor vascularization.

## 2. Norepinephrine and Epinephrine

Norepinephrine (NE) and epinephrine (E) belong to a family of catecholamines and are one of the best characterized stress neurohormones. NE is released primarily from the sympathetic nerves, while E is mainly secreted from the adrenal medulla. As the sympatho-adrenomedullary system is responsible for the body's fight-or-flight stress response, circulating levels of both catecholamines are increased during stress [17]. NE and E activate the same *α* and *β* adrenoreceptors (AR), which are widely distributed in all tissues.

Recently, NE and E have been implicated in stress-induced augmentation of tumor growth and progression. In an orthotopic model of ovarian carcinoma, the growth-promoting effect of stress was mimicked by a *β*-AR agonist, isoproternol, and blocked by its antagonist, propranolol [5, 6]. Similarly, activation of *β*-AR resulted in an increase in metastases in animal models of lung and breast cancer [18, 19]. In all of the above models, the growth-promoting effects of stress, as well as direct activation of *β*-ARs, was associated with a significant increase in tumor vascularization, while *β*-AR blockers reduced vessel density [5, 6]. Moreover, tumors derived from stressed animals had elevated levels of VEGF and other angiogenic factors, and the growth promoting actions of *β*-AR activation was reduced by blocking the VEGF pathway [5]. Thus, an increase in angiogenesis appears to be the main mechanism of growth-promoting effects of NE and E. Indeed, in various cancer cell types, such as ovarian cancer, colon cancer, melanoma, pharyngeal carcinoma, and multiple myeloma, activation of *β*-ARs present on tumor cells led to a dramatic increase in synthesis and release of angiogenic factors—VEGF, IL-8, and IL-6 [5, 16, 20–23]. These effects were mediated primarily via a *β*-AR-dependent increase in cAMP levels, which resulted in the activation of protein kinase A (PKA) and Src [5, 22]. Adrenergic stimulation has also been shown to increase the secretion of metalloproteases, MMP-2 and MMP-9, which further augment angiogenic and metastatic processes [21]. Interestingly, catecholamine-induced release of angiogenic factors from tumor cells can be further enhanced by its secretion from stromal cells, such as *β*-AR-positive tumor-associated macrophages [24, 25]. 

Although the stimulatory effects of NE and E on the release of angiogenic factors seem to be the major mechanism of their tumor-promoting actions, these neurotransmitters can also exert direct trophic effects on endothelial cells (ECs) through *α*-ARs. Phenylepinephrine, a non-vasoconstrictive *α*-AR agonist, has been shown to induce EC proliferation and migration as well as promote capillary formation. Interestingly, these effects were potentiated by hypoxia [26]. Since tissue ischemia is known to stimulate NE release from the sympathetic nerves [27], the direct angiogenic effect of NE can be significantly enhanced in hypoxic areas of tumors. 

Thus far, the results of experimental studies have confirmed that AR agonists exert strong stimulatory effects on tumor growth and agree that the release of angiogenic factors is the main mechanism of these actions. These discoveries open new possibilities of treatment with well-known drugs, such as antagonists of ARs. Some clinical data indicating decreased incidence of prostate cancer among cardiovascular patients treated with *β*-blockers corroborated the above findings [28, 29]. However, it is important to remember that the indirect, pro-angiogenic effect of AR agonists mediated by other angiogenic factors depends on the presence of these receptors on tumor cells, thus it can be tumor-specific. Moreover, the angiogenic actions of NE and E can be further modified by their direct effect on tumor cell proliferation and invasiveness, which in turn may differ among various tumors. In many cancer cell types, such as colon, ovarian, and prostate, these effects are stimulatory [11, 12]. However, adrenergic stimulation can also inhibit proliferation of some tumor cells, as shown in melanoma and neuroblastoma [30, 31]. In breast cancer, on the other hand, the reports are contradictory. The adrenergic agonists seem to increase motility of cancer cells but at the same time inhibit their proliferation [13, 32]. In agreement with these data, another clinical study indicated no effect of treatment with *β*-blockers on the risk of breast cancer among cardiovascular patients [33, 34]. Thus, the success of potential cancer therapy targeting ARs will depend on the type of tumor, its receptor expression pattern, and environmental factors, such as stress, which augment NE and E effects.

## 3. Dopamine

Dopamine (DA) is not only a precursor of NE and E but is also an important neurotransmitter in the brain acting via two types of receptors—D1 and D2. In the periphery, DA is synthesized in mesenteric organs as well as released from sympathetic neurons and adrenal medulla [17]. Levels of DA are elevated during stress, but rather than mediating the fight-or-flight response, as NE and E do, its role involves coping with stress [35]. DA also seems to have opposite than NE and E effects on tumor growth. It has been shown that administration of DA inhibits the growth of various tumors, such as stomach, breast, and colon cancers [14, 36]. Consistently, in mice lacking the DA transporter, which is normally responsible for uptake of this neurotransmitter, the elevated DA levels were associated with reduced growth of Lewis lung carcinoma [37]. In gastric cancer, the endogenous levels of DA were significantly lower than those in surrounding healthy tissue, indicating that the neurotransmitter acts as an endogenous tumor suppressant that needs to be inactivated to allow tumor progression [36]. 

The main mechanism of these growth-inhibitory actions of DA involves its direct antiangiogenic effect on ECs. In all animal models, treatment with DA led to a significant reduction in tumor vascularization [14, 36, 37]. DA has also been shown to block VEGF-induced EC proliferation, migration, and vascular permeability. Further studies revealed that DA, acting through its D2 receptors, enhances endocytosis of VEGF-R2 and decreases its membrane expression. This activity of DA interferes with VEGF signaling by reducing VEGF-induced phosphorylation of its VEGF-R2 and preventing the activation of downstream kinases—FAK and p42/44 MAPK [38, 39]. 

In addition to its effect on mature ECs, DA has also been shown to block VEGF signaling in endothelial progenitor cells (EPCs). As a consequence, DA not only inhibits trophic functions of VEGF in these cells but also blocks their recruitment from bone marrow. It has been shown that DA levels are decreased in the bone marrow of tumor-bearing mice, which facilitates EPC mobilization [40]. Since recent data strongly support a role for EPCs in the tumor vascularization, DA effect on EPC function may significantly contribute to its growth-inhibitory effect.

The role of DA in stress-induced changes in tumor growth and progression has not been characterized. It seems that DA is an endogenous inhibitory factor which requires inactivation for tumor growth, rather than sympathetic activation. However, in contrast to NE and E acting on specific tumors, DA effects appear to be more universal, influencing various tumor types, via its direct actions on ECs and EPCs. Thus, DA receptor agonists may become attractive antiangiogenic drugs in cancer therapy.

## 4. Neuropeptide Y

Neuropeptide Y (NPY) is a 36-amino-acid peptide coreleased with NE from sympathetic nerves. The actions of the peptide are mediated by multiple receptors-designated Y1–Y5 [41]. NPY is mainly known due to its anxiolytic effect in the brain and central regulation of food intake. In the periphery, NPY inhibits the release of NE after sympathetic stimulation and acts as a vasoconstrictor [41]. There is also a growing number of evidences that NPY is a growth factor for variety of cells. The peptide has been shown to stimulate proliferation of vascular smooth muscle cells and neuronal precursors, while the trophic effect of NPY on ECs revealed its angiogenic properties [42–47].

The main mechanism of NPY-induced angiogenesis involves its direct effect on ECs. The peptide stimulates proliferation and migration of ECs and promotes capillary tube formation, while in vivo, endogenous NPY facilitates vascularization of ischemic tissues [43, 46, 47]. These actions are dependent on endothelial nitric oxide synthase (eNOS) activation and, partially, on the VEGF pathway [46]. The angiogenic activities of NPY are mediated mainly by its Y2Rs, since NPY-induced angiogenesis is severely impaired in Y2R −/−  mice [48, 49]. 

Due to its angiogenic properties, NPY has been implicated in various pathological conditions associated with a deregulation of tissue vascularization, such as retinopathy, wound healing, atherosclerosis, and obesity [48, 50–52]. Recently, its role in tumor angiogenesis has also been shown. In malignancies originating from neuroendocrine tissues, such as neuroblastoma and Ewing's sarcoma, NPY released from tumor cells seems to be an essential factor involved in their vascularization. Antagonists to NPY receptors blocked the effect of both neuroblastoma and Ewing's sarcoma-conditioned media on EC proliferation. Consequently, treatment with exogenous NPY significantly increased vascularization of subcutaneous xenografts derived from both tumor cell types [10]. 

As in the case of NE and E, the angiogenesis-related growth-stimulatory actions of NPY are further modified by its direct effect on tumor cell growth and survival. For example, in neuroblastoma, the peptide stimulates proliferation of tumor cells via the same angiogenic Y2Rs, thereby further augmenting the growth of neuroblastoma xenografts. In contrast, in Ewing's sarcoma, NPY induces tumor cell apoptosis via Y1 and Y5Rs. As a result, exogenous NPY inhibits growth of Ewing's sarcoma xenografts in vivo, despite increase in their vascularization [10]. 

Although neuroendocrine tumors, which synthesize and release endogenous NPY, seem the most susceptible to tumor growth regulation by this peptide, NPY and its receptors have also been implicated in nonneuronal types of tumors. For example, peptide YY (PYY), which belongs to the same family of peptides and acts through the same receptors as NPY, has been shown to inhibit proliferation of breast and prostate cancer cells via Y4Rs and pancreatic cancer cells via Y2Rs [53–56]. Thus, these direct effects on tumor cell proliferation and survival are an important aspect of NPY actions in tumors and are often potent enough to overcome its angiogenesis-mediated growth-promoting effect.

Thus far, most of the studies addressing the role of stress in promoting cancer growth focus on the best known stress mediators—catecholamines and glucocorticoids. There are no studies directly linking NPY with stress-induced tumor growth and progression. However, systemic NPY levels are also upregulated during stress, particularly those intensive and prolonged in nature. Moreover, NPY is more stable than both NE and glucocorticoids. Hence, once stimulated, the elevated levels of NPY persist for a longer period of time [57]. The physiological role of NPY is to help cope with stress due to its central, anxiolytic effects [58, 59]. However, it has been shown that elevated peripheral circulating levels of NPY induced by intensive chronic stress can result in significant deleterious effects, such as enhanced atherosclerosis and diet-induced obesity, both of which are diseases associated with intensive tissue growth and upregulated angiogenesis [52, 60]. Thus, while high levels of NPY in the brain improve stress coping, chronically elevated levels of the peptide in the circulation can result in a variety of side effects. Whether enhanced tumor growth is one of them remains to be investigated.

## 5. Summary

**Figure 1:**
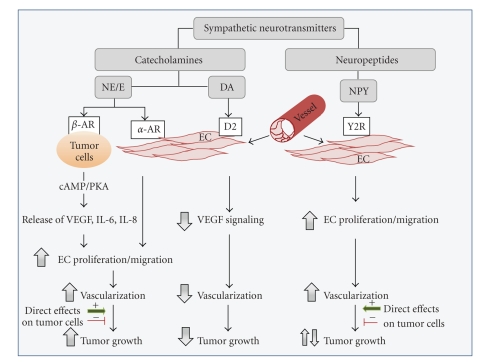
Activation of sympathetic neurons results in release of various neurotransmitters—catecholamines and neuropeptides. Norepinephrine (NE) and Epinephrine (E), belonging to a family of catecholamines, activate their *β*-adrenoreceptors (ARs) expressed on tumor cells and stimulate release of angiogenic factors, such as vascular endothelial growth factor (VEGF) and interleukins. Moreover, NE/E can directly induce endothelial cell (EC) proliferation and migration via their *α*-AR. Both of these processes lead to an increase in tumor vascularization. Adrenergic stimulation can also affect proliferation, survival, and invasiveness of cancer cells. This effect may be stimulatory or inhibitory, depending on tumor type. However, the proangiogenic actions of NE/E prevail over its direct effect on tumor cells. In consequence, adrenergic activation leads to an increase in tumor growth in most of the investigated tumor types. Another catecholamine, dopamine (DA), acts on its D2 receptors present on EC and interferes with VEGF signaling. As a result, dopamine reduces tumor vascularization and inhibits tumor growth. Neuropeptide Y (NPY), coreleased with NE from sympathetic nerves, directly stimulates EC proliferation and migration via its Y2Rs and increases tumor vascularization. However, NPY can also significantly alter the proliferation and survival of tumor cells. These direct actions of NPY on tumor cells are powerful enough to overcome its angiogenic activities. In consequence, the net effect of NPY varies in different types of tumors.

As summarized above, the discoveries of recent years provided a significant body of evidence confirming an important role of sympathetic neurotransmitters and, consequently, chronic stress in regulating of tumor vascularization (Figure 1). This research opens new avenues for developing novel therapeutics, as well as using already existing and well-characterized drugs, such as *β*-blockers and DA receptor agonists, in new clinical settings. This seems to be particularly important, since cancer diagnosis per se is usually a stressful event for the patient. However, careful consideration needs to be given to other actions of stress mediators, such as cancer-specific effects on tumor cells themselves, as well as changes in immune system, which can indirectly affect tumor development and progression. Finally, since patterns of neuro-hormonal activation vary with different types of stress [17], tumor exposure to particular stress mediators would vary, too. Thus, potential therapeutic value of modifying particular stress pathways may be dependent on a variety of factors.
